# Hereditary Folate Malabsorption Presenting With Pancytopenia in Two Siblings: A Case Report

**DOI:** 10.7759/cureus.89809

**Published:** 2025-08-11

**Authors:** Chaimae N'joumi, Ayad Ghanam, Hassnae Tkak, Abdeladim Babakhouya, Maria Rkain

**Affiliations:** 1 Pediatrics, Centre Hospitalier Universitaire Mohammed VI, Oujda, MAR; 2 Pediatric Medicine, Faculty of Medicine and Pharmacy, Mohammed I University, Centre Hospitalier Universitaire Mohammed VI, Oujda, MAR; 3 Pediatrics, Faculty of Medicine and Pharmacy, Mohammed I University, Mohammed VI University Hospital, Oujda, MAR; 4 Pediatric Gastroenterology, Centre Hospitalier Universitaire Mohammed VI, Oujda, MAR

**Keywords:** folinic acid, hereditary folate malabsorption, hypogammaglobulinemia, neurological symptoms, pancytopenia

## Abstract

Hereditary folate malabsorption (HFM) is a rare autosomal recessive disorder caused by mutations in the SLC46A1 gene, leading to impaired intestinal and central nervous system folate transport. We present two male siblings with clinical features suggestive of HFM. The first infant exhibited pancytopenia, diarrhea, hypogammaglobulinemia, and neurological regression due to delayed diagnosis and treatment discontinuation, resulting in a fatal outcome. The second sibling was diagnosed early based on clinical suspicion and family history and showed favorable progress after timely parenteral folinic acid therapy. This report underscores the importance of early recognition, the limitations of genetic access in low-resource settings, and the critical role of parenteral folinic acid in preventing irreversible complications.

## Introduction

Hereditary folate malabsorption (HFM) is a rare autosomal recessive disorder first described by Luby et al. in 1961 [1]. It results from mutations in the SLC46A1 gene, which encodes the proton-coupled folate transporter (PCFT) responsible for folate absorption in the intestine and transport into the central nervous system [2]. Newborns appear normal at birth due to maternal folate supply, but symptoms emerge within weeks to months as stores deplete. Clinical features include diarrhea, failure to thrive, pancytopenia, and neurological deficits such as seizures and developmental delays due to low cerebrospinal fluid folate [3]. Immunodeficiency may also occur, predisposing to opportunistic infections such as *Pneumocystis jirovecii *pneumonia [3]. Diagnosis requires high suspicion, especially in infants with multisystem involvement and poor response to oral folate. Lifelong treatment with folinic acid, preferably intramuscular, is essential [4].

We present the case of two male siblings with suspected HFM who developed life-threatening pancytopenia. While genetic testing was unavailable, the diagnosis was strongly supported by their clinical features, family history, and laboratory findings. One sibling succumbed to complications from delayed diagnosis and interrupted treatment, whereas the other was promptly diagnosed and successfully treated with parenteral folinic acid. This report aims to draw attention to the importance of early diagnosis and continuous management, particularly in resource-limited settings where genetic confirmation is not readily available, and to illustrate the variability of outcomes that may occur among affected siblings.

## Case presentation

Patient 1 (index case)

**Figure 1:**
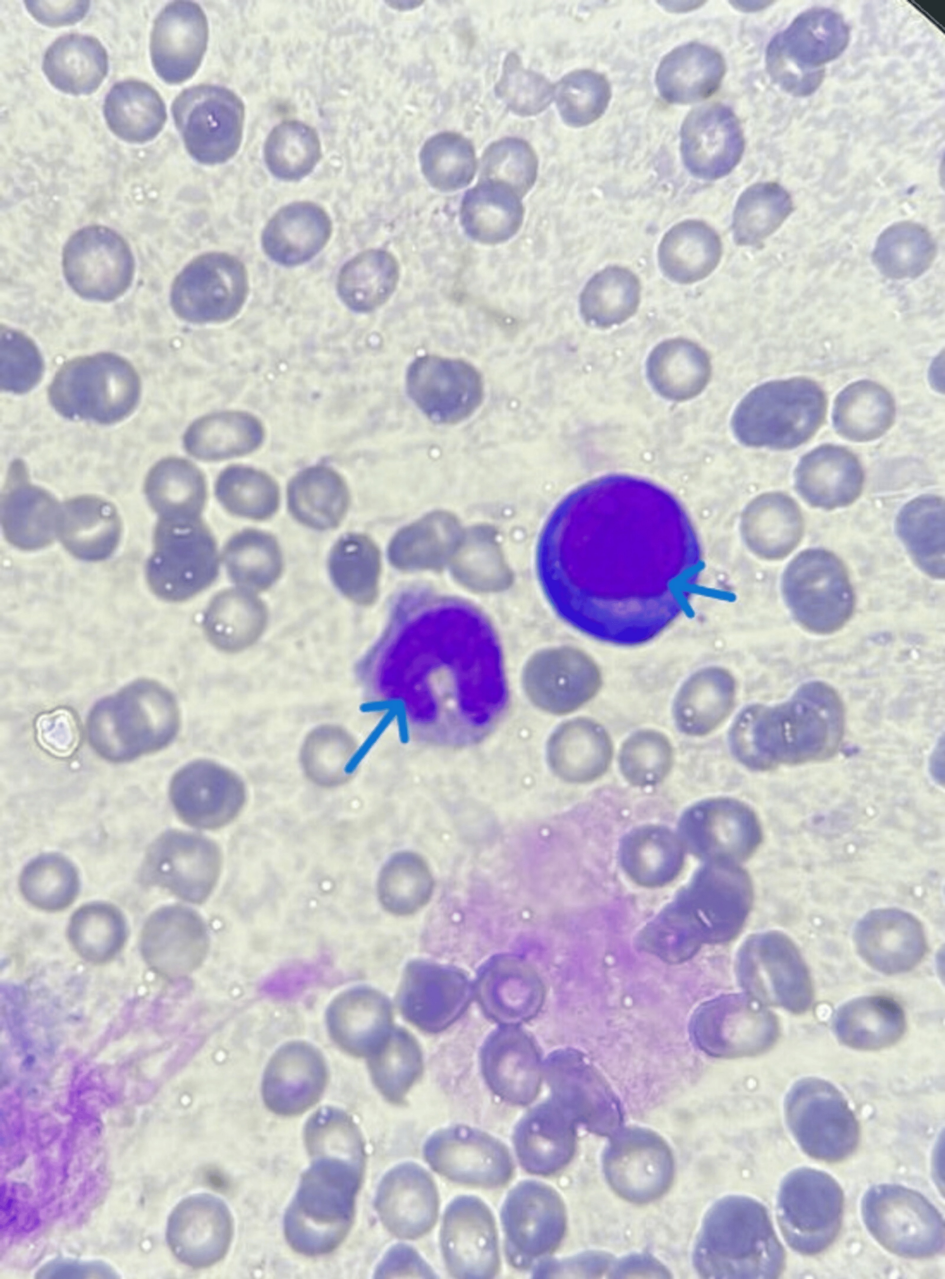
Megaloblastic features (megaloblast and metamyelocyte) on bone marrow smear of the index case

A previously healthy male infant was admitted at two months of age for evaluation of pallor, hypotonia, feeding difficulties, and persistent diarrhea lasting for one month. On examination, he was conscious and pale with a capillary refill time of less than three seconds. Anthropometric measurements were within normal limits. Conjunctival pallor was observed, and no hepatosplenomegaly was detected on examination. Initial laboratory studies revealed pancytopenia characterized by macrocytic anemia, leukopenia with neutropenia, and thrombocytopenia. Hemolysis workup demonstrated decreased haptoglobin and elevated lactate dehydrogenase. Ferritin levels were increased. Immunoglobulin profiling revealed hypogammaglobulinemia. Liver and renal function tests, electrolytes, and lipid profile were within normal limits (Table 1). Bone marrow aspiration revealed marked megaloblastic changes characteristic of folate deficiency, with cellular gigantism demonstrated by megaloblasts and enlarged metamyelocytes featuring band-shaped nuclei (Figure 1). Serum folate was profoundly decreased, with normal vitamin B12 levels. Plasma homocysteine was elevated.

**Table 1:** Biological parameters of the index case at first admission

Parameter	Patient’s value	Normal range
Hemoglobin (Hb)	4.40 g/dL	10.5-14.0 g/dL
Mean Corpuscular Volume (MCV)	99,500 fL	80,000-98,000 fL
White Blood Cells (WBC)	2,620,000/µL	4,000,000-10,000,000/µL
Neutrophils (PNN)	550,000/µL	1,500,000-7,000,000/µL
Platelets	63,000,000/µL	150,000,000-400,000,000/µL
Ferritin	416,000 ng/mL	50,000-200,000 ng/mL
Vitamin B9 (Folate)	< 2,200 ng/mL	2,340-17,560 ng/mL
Vitamin B12	241,000 pg/mL	187,000-883,000 pg/mL

The patient was started on intravenous (IV) folinic acid therapy at a dose of 2 mg/kg/day, which led to initial hematologic improvement and normalization of immunoglobulin levels, followed by oral folic acid supplementation. Nutritional assessment confirmed adequate dietary intake, exclusive infant formula feeding. Maternal folate status and socioeconomic factors were unremarkable. However, after initial improvement, the infant was lost to follow-up. According to caregiver reports, oral folate supplementation was discontinued, and no further clinical or laboratory monitoring occurred. At 11 months, the patient was readmitted with generalized tonic-clonic seizures (reported by the mother), psychomotor regression including loss of acquired milestones, and feeding difficulties. Clinical examination showed hypotonia and mild dehydration; anthropometrics remained within appropriate ranges. Repeat laboratory tests confirmed persistent folate deficiency and elevated homocysteine, while liver and renal functions and inflammatory markers were normal. Cerebrospinal fluid (CSF) folate measurement was unavailable. Brain MRI and EEG were unremarkable. Other metabolic, infectious, and structural causes for seizures were excluded. Infectious workup included blood cultures, CSF analysis with bacterial culture, urine culture, and inflammatory markers, such as C-reactive protein and procalcitonin. Metabolic investigations included serum electrolytes, blood glucose, liver and renal function tests, blood gas analysis, ammonia, and lactate levels. Folate deficiency was determined to be the likely cause. Despite aggressive supportive care - including parenteral folinic acid, blood transfusions, and broad-spectrum antimicrobial, antiviral, and antifungal therapies, as well as nutritional support - the patient developed pneumonia, leading to respiratory failure and death.

Patient 2 (sibling)

The mother, pregnant at the time of the index patient’s hospitalization, gave birth to a male infant who was evaluated at two months of age for pallor, oral candidiasis, and feeding difficulties. There was no history of vomiting, seizures, or fever. Family history was significant for the death of the older sibling at 11 months due to severe folate deficiency complications. On examination, the infant was pale and mildly hypotonic with oral thrush. Growth parameters were within normal percentiles. Cardiopulmonary and abdominal exams were unremarkable. Neurological examination showed generalized hypotonia with preserved reflexes and spontaneous movements. Laboratory evaluation demonstrated pancytopenia with macrocytic anemia and megaloblastic features. Serum folate was markedly decreased; vitamin B12 and other biochemical parameters, including liver and renal functions and inflammatory markers, were normal (Table 2). CSF folate levels were also measured and found to be markedly low. Maternal folate and dietary intake were adequate. Given the combination of characteristic clinical features, low serum and CSF folate levels, and a significant family history, HFM was strongly suspected. Although genetic confirmation was not available owing to limited resources, the diagnosis was considered highly likely based on these concordant criteria. The infant was started on exclusive intramuscular folinic acid therapy, deliberately avoiding oral supplementation to bypass intestinal malabsorption during critical neurodevelopmental stages. Hematologic recovery was achieved within two weeks, and at six months of follow-up, the infant shows stable blood counts and age-appropriate neurodevelopmental progress.

**Table 2:** Biological parameters of the sibling case at admission

Parameter	Patient’s value	Normal range
Hemoglobin (Hb)	4.70 g/dL	10.5-14.0 g/dL
Mean Corpuscular Volume (MCV)	99,500 fL	80,000-98,000 fL
White Blood Cells (WBC)	2,850,000/µL	4,000,000-10,000,000/µL
Neutrophils (PNN)	160,000/µL	1,500,000-7 000,000/µL
Platelets	20,000,000/µL	150,000,000-400,000,000/µL
Ferritin	472,400 ng/mL	50,000-200,000 ng/mL
Vitamin B9 (Folate)	< 2,200 ng/mL	2,340-17,560 ng/mL
Vitamin B12	375,000 pg/mL	187,000-883,000 pg/mL
Cerebrospinal Fluid (CSF) Folate	< 1,500 nmol/L	~100,000 nmol/L

## Discussion

Folate (vitamin B9) is a water-soluble vitamin essential for DNA/RNA synthesis, methylation reactions, and amino acid metabolism, particularly homocysteine [2]. In its active form, tetrahydrofolate, it supports purine and pyrimidine synthesis and is critical for hematopoiesis and neurological development [5]. Folate is primarily absorbed in the duodenum and jejunum via the proton-coupled folate transporter (PCFT), encoded by the SLC46A1 gene [5]. PCFT also facilitates folate transport across the blood-cerebrospinal fluid barrier at the choroid plexus. Mutations affecting PCFT function lead to a severe yet treatable form of folate malabsorption [2].

HFM is a rare autosomal recessive disorder caused by mutations in SLC46A1, impairing both intestinal folate uptake and transport into the CNS, resulting in systemic and central folate deficiency [5]. Affected infants are typically born with sufficient folate stores, but deficiency develops early - most often between two months and one year of age - due to impaired absorption from breast milk or formula [6].

HFM is extremely rare, with fewer than 100 cases reported worldwide to date, mainly as isolated case reports originating from approximately 45 families [6]. The true prevalence is likely underestimated, as affected infants may die before the diagnosis is made, particularly in regions with high consanguinity and limited access to healthcare [6].

Clinical presentation is highly heterogeneous, even among individuals carrying the same mutation, ranging from isolated hematologic abnormalities to severe multisystem involvement, reflecting folate’s roles in hematologic, gastrointestinal, immunologic, and neurologic systems. The most consistent hematologic finding is megaloblastic anemia due to defective DNA synthesis. Pancytopenia is also common, with anemia, leukopenia, and thrombocytopenia, as seen in our patient [2]. These abnormalities typically improve rapidly with parenteral folate therapy, underscoring the importance of early diagnosis [3].

Gastrointestinal symptoms include chronic diarrhea, oral ulcers, and feeding difficulties, which may lead to malnutrition and failure to thrive [7]. In our case, the patient had feeding refusal, oral lesions, and diarrhea-findings consistent with the disease's gastrointestinal involvement.

Immune dysfunction in HFM results from impaired lymphocyte DNA synthesis, often leading to hypogammaglobulinemia. This contributes to increased susceptibility to infections, especially of the respiratory tract, with organisms such as *Pneumocystis jirovecii* and *Cytomegalovirus *occasionally reported. In some cases, infants may die of infections before HFM is recognized [8]. Our patient experienced recurrent respiratory infections, which resolved after initiating folate therapy, highlighting the reversibility of immune dysfunction with treatment [8].

Neurologic symptoms - such as developmental delay, seizures, movement disorders, and cognitive impairment - are frequent in untreated HFM. These result from folate deficiency in the CNS, which disrupts myelination and neurotransmitter synthesis [5]. Neuroimaging may show delayed myelination, cerebral atrophy, or basal ganglia calcifications, though findings are variable [6]. In our case, MRI was normal despite recurrent seizures and psychomotor regression, illustrating the heterogeneity of neurologic involvement and the need for early treatment to prevent irreversible damage.

Diagnosis of HFM relies on identifying low serum and cerebrospinal fluid folate levels, often with elevated plasma homocysteine [2]. Although genetic confirmation via SLC46A1 sequencing is ideal, it is not always available in low-resource settings [6]. According to established diagnostic criteria, HFM may be diagnosed in the presence of anemia, impaired folate absorption, and markedly decreased CSF folate levels, even without molecular confirmation [6]. Our patient fulfilled these criteria, displaying macrocytic anemia, very low serum and CSF folate, elevated homocysteine, lack of response to oral folate (as evidenced by poor response to oral folate in the older sibling), a strong family history, and rapid response to parenteral folinic acid. Therefore, despite the absence of genetic testing, sufficient diagnostic certainty was reached to justify prompt treatment. Highlighting this diagnostic approach is particularly relevant for low-income countries, where HFM is more likely to occur due to high rates of consanguinity, yet genetic confirmation is often unavailable, to encourage clinicians to consider this diagnosis based on clinical and biochemical criteria and initiate life-saving therapy without delay.

Treatment focuses on correcting systemic and CNS folate deficiency as early as possible to prevent complications. Delays in restoring CSF folate can lead to lasting neurological damage [9]. Folinic acid (5-formyltetrahydrofolate) is preferred, as it bypasses the defective PCFT transporter. Folic acid should be avoided due to its ineffectiveness and potential interference with folate metabolism [6]. Intramuscular folinic acid is often required, especially to restore CSF folate levels and support neurological recovery [10]. Oral folinic acid may improve hematologic and immune features, but intramuscular doses (typically 0.5-3 mg/kg/day) are more effective in severe cases [5]. Levofolinic acid, the active isomer, may provide additional benefits where available [10]. In our patient, clinical and hematologic recovery following parenteral folinic acid highlighted the effectiveness of early targeted treatment.

## Conclusions

HFM is a rare but treatable disorder that requires early recognition to prevent severe hematologic and neurological complications. Our report highlights the critical importance of timely diagnosis and initiation of parenteral folinic acid therapy to achieve favorable outcomes. In resource-limited settings, where genetic testing is often unavailable, clinicians should rely on clinical and biochemical criteria to promptly identify and manage affected patients. Improving access to diagnostic tools and awareness of this condition is essential to reduce morbidity and mortality, especially in regions with high consanguinity rates.
